# Sourdough Biotechnology Applied to Gluten-Free Baked Goods: Rescuing the Tradition

**DOI:** 10.3390/foods10071498

**Published:** 2021-06-28

**Authors:** Laura Ramos, Alicia Alonso-Hernando, Miriam Martínez-Castro, Jose Alejandro Morán-Pérez, Patricia Cabrero-Lobato, Ana Pascual-Maté, Eduardo Téllez-Jiménez, Jorge R. Mujico

**Affiliations:** 1 Facultad de Ciencias de la Salud, Universidad Isabel I, 09003 Burgos, Spain; laura.ramos@cajal.csic.es (L.R.); miriam.martinez@ui1.es (M.M.-C.); jaleomp@gmail.com (J.A.M.-P.); patricia.cabrero@ui1.es (P.C.-L.); ana.pascual@ui1.es (A.P.-M.); eduardo.tellez@ui1.es (E.T.-J.); jrmujico@gmail.com (J.R.M.); 2 Unidad de Citometría de Flujo y Separación Celular, Instituto Cajal, CSIC, 28002 Madrid, Spain

**Keywords:** celiac disease, gluten-free, food additives, sourdough, microbiota, lactic acid bacteria

## Abstract

Recent studies suggest that the beneficial properties provided by sourdough fermentation may be translated to the development of new GF products that could improve their technological and nutritional properties. The main objective of this manuscript is to review the current evidence regarding the elaboration of GF baked goods, and to present the latest knowledge about the so-called sourdough biotechnology. A bibliographic search of articles published in the last 12 years has been carried out. It is common to use additives, such as hydrocolloids, proteins, enzymes, and emulsifiers, to technologically improve GF products. Sourdough is a mixture of flour and water fermented by an ecosystem of lactic acid bacteria (LAB) and yeasts that provide technological and nutritional improvements to the bakery products. LAB-synthesized biopolymers can mimic gluten molecules. Sourdough biotechnology is an ecological and cost-effective technology with great potential in the field of GF products. Further research is necessary to optimize the process and select species of microorganisms robust enough to be competitive in any circumstance.

## 1. Introduction

Celiac disease (CD) is an immune-mediated systemic disease, caused by gluten and related prolamins intake in genetically susceptible individuals. CD can only be treated by a lifetime adherence to a gluten-free (GF) diet, by removing wheat, barley, rye, oats, and their hybrids from the daily food intake [1,2,3].

When CD patients continuously ingest gluten, the small intestine mucosa is damaged by an increased number of lymphocytes and can evolve into villus atrophy and crypt hyperplasia [3]. The sustained consumption of gluten in these patients, even at trace levels, maintains the pathology and the intestinal damage, although there are no apparent clinical symptoms. The damage is accompanied by a malabsorption of nutrients that can lead to chronic diarrhea, abdominal distension, and reduced physical growth (the classic CD triad). Although CD has been traditionally considered as a gastrointestinal disease, nowadays, it is classified as an autoimmune-mediated systemic disease, affecting several organs and tissues [4].

The worldwide prevalence of CD is around 1.4% [5], with a heterogeneous distribution, that mainly affects Caucasians, and is more frequent in women than in men (in a ratio of approximately 2.8:1) [5,6]. The major problem related to this disease are the undiagnosed cases, since they can present atypical or no symptoms at all. It is estimated that 83% of celiac patients are not conscious of their disease [7], a percentage that increases up to 90% in pediatric patients [8], a phenomenon known as the “celiac iceberg” [9].

Commercialized GF products usually present technologically associated drawbacks related to the elasticity and cohesion of the dough, two properties provided by gluten proteins. As gases produced during fermentation are difficult to retain, they also show less volume and fluffy texture. These GF products are clearly inferior compared to their gluten-containing (GC) counterparts, since they are worse at a sensorial level, present low nutritional quality, and are more expensive [10,11]. The development of high-quality GF bakery products is a challenge for the food science and technology community, which is going through two different approaches: (i) from the technological perspective, using aeration by high pressure, flour pretreatment with ultrasounds, partial baking with freezing cycles, hydrothermal and extrusion treatments, etc.; and (ii) from the scientific perspective, with modified formulations, such as using additives–adjuvants, and/or the sourdough-based biotechnology [12,13,14,15].

Sourdough is a mixture of flour and water that is fermented by the action of microorganisms. The fermentation process can be spontaneous or directed by the addition of commercial starter cultures. Sourdough microbiota is composed by different lactic acid bacteria (LAB) and yeasts, in a ratio of approximately 100:1; both types of microorganisms can be naturally found in the cereal grains (and, consequently, in their flours), or provided by the “house microbiota” present in the physical environment where sourdough is made [16]. The main function of LAB is the acidification of the dough, producing chemical, metabolic and enzymatic modifications, whereas the main function of yeast is carbon dioxide (CO_2_) production.

In the elaboration of baked goods, there is a tendency towards the recuperation of sourdough fermentation due to its numerous beneficial properties caused by the fermentation and acidification of dough by the native microbiota. Table 1 presents some sourdough properties that improve the quality of bakery products. These beneficial properties include organoleptic [17], nutritional [18,19], and functional [20] improvements, as well as an extension of the shelf life of baked goods [21]. Recent studies suggest that these positive effects may be translated to the development of new GF products, solving their low-quality properties.

The general objective of this paper is to analyze the scientific evidence regarding the production of GF baked goods (mainly bread), and to present the latest knowledge about sourdough biotechnology. The use of additives or adjuvants in GF bakery products, alone or in combination with sourdough biotechnology, the autochthonous LAB and yeast naturally present in GF sourdoughs, and the microorganisms that synthesize gluten-like molecules that thereby improve the bakery products, will be also described.

A bibliographic search was performed between September and December of 2020 in Scopus, ScienceDirect, PubMed/Medline, and FSTA (Food Science & Technology Abstracts) databases. The following keywords and Boolean operators, both in Spanish and in English, were used: (adjuvant OR additive OR hydrocolloid OR protein OR enzymes OR emulsifiers) AND gluten-free bread; (lactic acid bacteria OR LAB OR sourdough OR yeast OR microbiota OR microbiome OR ecology OR biota) AND gluten-free NOT human; (lactic acid bacteria OR LAB OR exopolysaccharides OR EPS OR sourdough AND gluten-free). The search was restricted to studies containing the terms of reference in both the title and the abstract, using the query [TIAB] (TITLE AND ABSTRACT). The search was limited to studies published during the last 12 years, including research papers, meta-analysis, reviews and/or systematic reviews, books, or thesis. Then, a total of 92 studies that met these criteria which analyze ingredients, or final products mainly based on GF cereals (rice, corn/maize, millet, sorghum) and/or pseudo-cereals (buckwheat, quinoa, amaranth, teff) were finally selected for this review.

## 2. Technological Aspects of Using Additives and Adjuvants in Gluten-Free Baked Goods and Joint Contributions with Sourdoughs

Hydrocolloids, proteins, enzymes, and emulsifiers are the most-used additives and adjuvants in the preparation of GF bakery products. Its widespread use implies that they are also common ingredients in GF bread formulations that include sourdoughs, both in already developed products or products under research. The technological advantages provided by these compounds are briefly described in the following subsections.

### 2.1. Hydrocolloids

Hydrocolloids are a group of water-soluble polymers that are used in the formulation of GF doughs because they improve the properties of the final product in terms of structure, volume, texture, and palatability, as well as shelf-life extension. With very different chemical structures, they can be classified according to their origin, from: (i) some species of seaweed, such as agar–agar or carrageenan; (ii) plant tissue extracts, such as pectin, β-glucan or inulin; (iii) plant exudates, such as gum arabic (extracted from the resin of some varieties of acacia); (iv) different viscous plant substances (also called mucilages), such as guar gum or psyllium; (v) exopolysaccharides (EPS) of microbial origin, such as xanthan gum (synthesized by *Xanthomonas campestris*), or gellan gum (synthesized by *Sphingomonas elodea*), brought naturally from the addition of sourdoughs to the GF batter or artificially included on it; and (vi) cellulose-derived molecules, such as methylcellulose (MC), carboxymethylcellulose (CMC), or hydroxypropyl methylcellulose (HPMC) [22,23].

This group of compounds can mimic, to some extent, the viscoelastic properties of gluten in the dough. This is due to its capacity to interact with water and form a network-like structure (gel properties) that increases the viscosity of the mixture, as well as the capacity to retain the CO_2_ produced during fermentation. They also stimulate the gelatinization of starches during baking, reducing the crystallization of amylopectin (starch retrogradation), and keeping products fresh for longer periods of time [24].

Hydrocolloids are the most widely used additives in the GF products’ industry. Their ability to bind water in doughs (increasing viscosity and providing gel characteristics, which somehow mimics gluten technological properties), was already discovered in the 1950s. In this context, and from a scientific point of view, hydrocolloids are the most studied additives. There is a great number of experimental research studies that have analyzed how these molecules behave in different mixture/dough matrices.

Although the same type of hydrocolloid is used, results published in the literature are divergent, since the added concentration range is another variable to be considered (Table 2). It is usual to employ concentrations ranging from 0.3 to 5%, always selecting the lowest concentration with the best results. Additives are expensive and can provide (based on the concentration they are used), strange and undesirable flavors to the final product. In addition, the relationship between concentration and technological improvement is not directly proportional: once an optimum concentration is achieved (based on each additive and each dough), increasing the amount of additive does not lead to further improvement of the final product, and a collapse of the dough may occur, thus decreasing the improvement/s obtained [11].

There are different flours (mixed, or not), hydrocolloids (mixed, or not, at different concentrations), and other substances (such as water, salt, sugar, honey, butter, milk, whey, etc.) that may be present in the dough. Water can be highlighted among all of them, due to its technological impact (it is fundamental in the final product and must be also optimized). GF dough generally requires greater amounts of water, ranging from 50 to 218%, and this proportion has an influence on the other parameters. It even affects baking: more hydrated doughs need baking containers (because of their lower densities), and the size, dimensions, and material of these containers also influence the final baked good.

Longer baking times are also needed to remove this excess of water, which requires lower baking temperatures so that, for example, the crust is not excessively browned. Consequently, there are many parameters to consider when choosing the best ingredients, processes, and additives to obtain the desired result. All this complexity is reflected in the papers selected for this part of the review (Table 2) and, to some extent, it explains the disparity and lack of homogeneity between the obtained results.

The most used hydrocolloids in GF bakery products, and the ones that seem to work better, are HPMC and xanthan gum. Both HPMC and the rest of the cellulose derivatives employed as additives usually come from plant sources, although the so-called bacterial cellulose (BC) is also described, a related molecule synthesized by bacteria of the genus *Gluconacetobacter*, especially *G. sansei*. Recent studies have concluded that the production cost of this BC is so high, and the recovery yield so low, that it cannot be applied at an industrial scale [33].

In the paper by Hager and Arendt [27], included in the review published by Capriles and Arêas in 2014 [12], the use of these two hydrocolloids (HPMC and xanthan gum) was reviewed, reaching the conclusion that HPMC has positive effects on formulations with teff and corn flours, negative effects when rice flour is used, and no changes for buckwheat flour; no conclusions were reached when the effect of xanthan gum was studied.

In the same study [27], a very little amount of hydrocolloid (around 0.14%) was needed when adding xanthan gum to buckwheat flour to obtain optimal results, determined by a higher loaf volume and softer crumb. To obtain the same results in corn flour, a higher concentration of HPMC (1.77%) was needed. During a third part of the same study, to check if the effects of these hydrocolloids were synergistic (potentiated), or could present some antagonism, teff flour and different ratios of HPMC and xanthan gum were tested. To reach the established objectives, it was necessary to slightly increase the concentration of HPMC compared to the one used alone (up to 2%), but the amount of xanthan gum to be added was very small, around 0.04% (70% less than used alone).

Schober et al. [34] obtained an improvement of sorghum bread quality with HPMC (2%) alone, but also showed that a previous sourdough fermentation of the total sorghum flour in combination with HPMC (2%), could solve some technological problems and lead to a superior quality sorghum bread.

Campo et al. [35] worked with GF bread formulas containing different combinations of teff flour (10%) and commercial dried cereal sourdoughs (rice or buckwheat, 15%) or *Lb. helveticus* fresh sourdough (15%), all of the batches including 0.75% HPMC, as a standardized concentration of this hydrocolloid. Bread with a combination of teff (10%) and rice-based sourdough achieved the best sensory results in terms of flavor [35].

Dermirkesen et al. [36] added different hydrocolloids (xanthan gum, carob gum, guar gum, and HPMC) to rice flour and, in their experimental conditions, the best combination was obtained by mixing xanthan and guar gums (paper included in the review published in 2016 by Mir et al. [25]). However, in another study also using rice flour, the highest loaf volumes were showed when CMC and HPMC were combined [37], included in the review published in 2016 by Mir et al. [25].

Keeping in mind that all these additives must be declared on the label and make the final product more expensive, research about other compounds providing more benefits than only technological is being encouraged—for example, those with added nutritional properties, such as inulin or β-glucans [38].

Regarding inulin, the results were again different between studies. Gularte’s group employed inulin in GF baked goods, and the results were not satisfactory: compared with control, it did not improve final loaf volume and, in addition, its use was counterproductive, increasing crumb firmness and decreasing elasticity ([39], study included in the review published in 2014 by O’Shea et al. [28]). In contrast, although Korus et al. obtained positive results by adding only 4% inulin, undesirable crust wrinkles appeared when inulin was increased up to 8% ([40], work included in the review published in 2016 by Drabińska et al. [29]).

The conclusion reached after reviewing all these studies, which is not only applicable to hydrocolloids but also to the use of any additive in GF baked goods, is that no correlation between the variables is found, and each case must be analyzed and assessed individually. The effect of additives, or adjuvants in the dough depends on the type and concentration of the additive, its interaction with other additives/ingredients, and any other technological parameter of the process. Besides the scientific literature results, the selection of the best compound/s to achieve a specific technological property should also consider if the substance is previously authorized as a food additive within regulations from the specific regions or countries and the individual restrictions to its use that would apply in every case.

### 2.2. Proteins

The use of proteins in GF baked products responds to a double objective: firstly, the nutritional value is increased (providing higher levels of protein and essential amino acids) and, secondly, some of these proteins (with the capacity for stabilizing foams and emulsions), can mimic gluten technological properties, improving the organoleptic characteristics, and leading to higher quality products.

The most used proteins come from egg and milk; proteins from soybean and other cereals and/or pseudo-cereals are also widely used:

- Egg proteins (helped by the lecithin present in the yolk), act as foaming and emulsifying agents, and they are capable of stabilizing emulsions. These properties will improve the dough structure and gas retention, providing a softer crumb with more uniformly distributed alveoli. In addition, egg is a food with a very interesting nutritional profile, considered as a good source of high biological value proteins, fats, vitamins, and minerals (especially iron).
- The most-used milk proteins are casein, caseinates, and whey proteins. These proteins have gluten-like functional and technological properties, capable of creating cross-linked networks, and with a high capacity for swelling and water retention. Regarding GF bread, milk proteins contribute to Maillard reaction (between amino acids and reducing sugars), improving texture, roasted flavors and, mainly, both color and aroma crust quality.
- Although soybean is a protein-rich food, it is deficient in sulfur-containing amino acids, such as the essential amino acid methionine. It is used as a functional food to increase the nutritional value of GF bakery products, since it contains bioactive compounds such as isoflavones. Due to its technological properties, soybean also has a positive impact on the quality of the final product, by improving crumb, volume, water retention, and sensory assessment.

When proteins are reviewed (Table 3), each individual case must be studied, analyzing the type of flour, the protein that has been used, and the manufacturing process.

One disadvantage of using proteins is that some of them (such as from milk, egg, and soybean), are classified as allergens, not being well accepted by patients with allergies, intolerances, and/or sensitivities to these foods. On the other hand, if milk-derived proteins are used, they must be low in lactose, since CD patients may have a secondary intolerance to this disaccharide, due to lactase deficiency, because of their villus atrophy [42].

### 2.3. Enzymes

Enzymatic technology is widely used in GC bakery for improving dough properties and final quality. Among all the used enzymes, highlights include: (i) amylase, breaking complex carbohydrates into sugars that can be used as substrates; and (ii) proteases, hydrolyzing gluten and being used, for example, in the production of cookies, providing a better malleable dough.

In GF bakery, enzymes are used to modify the proteins present in the dough into others capable of mimicking the action of the gluten proteins they lack (Table 4). The most frequently used enzymes are:

- Enzymes that modify starches, such as amylase and cyclodextrin glycosyltransferase; the latter degrades starch and produces dextrin that has been experimentally proven to increase the solubility of hydrophobic proteins, which in turn increases CO_2_ retention, providing a bigger loaf volume and a better texture [12,29,45]. Schober et al. indicated that bacterial α-amylase is used to supply sugars in the sourdough fermentation step, and also exerts an anti-staling effect in GF starch breads, so they included 0.01% of this enzyme in their sorghum sourdough formula [34].
- Enzymes that crosslink, or connect proteins, such as transglutaminase (TGase) and gluco-oxidase (GO). These enzymes, which catalyze protein polymerization and crosslinking reactions, can create a kind of network or mesh, such as the three-dimensional structure provided by gluten, that improves CO_2_ retention [12,30,46].
- Proteases that hydrolyze the peptide bonds of the proteins. This property can improve texture and final quality of rice-flour-based breads [12,30]. Additionally, proteolysis that occurs during the sourdough fermentation process could prevent interferences between protein aggregation upon baking and the starch gel, which seems to be desirable in GF sorghum breads [34].

To deal with the lack of gluten of these GF baked products, enzymes are perhaps the least used additives because, among other reasons, it is a very recent research area. Moreover, enzymes work at very low concentrations and what initially seems to be an advantage makes that slight increase of enzymes produce huge protein changes with unexpected results in the final products (such as loaves with low volume and very hard crumb) [44].

Renzetti et al.’s [43] paper included in the review published in 2017 by Naqash et al. [15] investigated the use of TGase in GF bakery without any other adjuvant addition. Their conclusion was that TGase could improve the functionality of GF flours, obtaining positive results in buckwheat and whole rice breads, also being of interest to continue researching the use of TGase together with other additives. Mohammadi et al.’s [49] paper included in the review published in 2017 by Naqash et al. [15] studied the addition of TGase together with guar gum in rice flour; the combination that better worked in their conditions was 1 U/g of TGase and 20 to 30 g/kg of guar gum (as more TGase was added, the hardness of the crumb was increased).

The use of enzymes in dough is widespread because of its technological potential for modifying proteins. Moore et al.’s [45] paper included in the review published in 2014 by Capriles and Arêas [12] tested increasing concentrations of TGase with the addition of proteins from different sources (egg, milk, soybean, cereals, etc.), without finding a clear correlation. The improvement of the dough was based on the flour, TGase concentration, and type of protein used. However, Storck et al.’s [46] paper included in the review published in 2014 by Capriles and Arêas [12] optimized the use of TGase and protein in their rice-flour-based model. The mixture of 1.35 U of TGase for each gram of protein (albumin+casein), together with 0.67% albumin and 0.67% casein, was the combination that provided the highest volume, and a crumb with more alveoli and less hardness.

However, recent observations have established a possible association between the increased use of microbial TGase in food processing and the surge in incidence of celiac disease [47].

### 2.4. Emulsifiers

Emulsifiers are substances with an amphiphilic nature, which means that one side of the molecule is hydrophilic (water soluble) and the other side is hydrophobic (water insoluble). This dual nature allows emulsifiers to stand between two immiscible phases, connect them, reduce surface tension, and form a stable, homogeneous, and fluid emulsion. The most frequently used emulsifiers are:

- Soy lecithin, a plant origin product, which is extracted from soybeans. It has a very high concentration of phospholipids that contribute to dough extensibility, and flour hydration properties.
- Mono- and di-glycerides of fatty acids (E–471) [48] have the property of softening the dough, facilitating mixtures at an industrial level, thus achieving a crumb with more alveoli and a larger final volume. They also decrease starch retrogradation, which improves the shelf life of bakery products (especially pastries).
- Esters of mono- and di-glycerides fatty acids (E–472a–E47f) [48], are mainly used in the preparation of bread, since they provide a better “body” to the dough (an excessively liquid dough is an important defect of the GF products); this equates to a firmer dough with greater gas retention, and both texture and final volume improvements. These emulsifiers also contribute to an increased shelf life of bakery products.

There are few studies where emulsifiers are used as a separate category of additives (Table 5). This is because many additives with emulsifying properties are classified as hydrocolloids, proteins, or enzymes (described in the previous subsections). It is worth highlighting those studies where DATEM^®^ (a commercial emulsifier) is investigated.

## 3. Sourdough Biotechnology

As previously described, sourdough can be considered as a specific ecosystem of LAB and yeasts that coexist in a flour–water matrix. Sourdough biotechnology could have a prehistoric origin, since ancient loaves have been found in Egyptian tombs, and wheat sheaves in human settlements dating from over 8000 years ago [51].

The elaboration of bread with these leavening microorganisms was abandoned in the second half of the 20th century, because of changes in food habits and the availability of commercialized pressed yeast. At that time, the food industry was consolidated, refrigerators arrived for domestic homes, and a boom of processed and ready-to-eat food products started to be sold in supermarkets.

Furthermore, important social changes started to happen, such as female economic independence, changes in eating behaviors (e.g., eating outside the home), etc. that have reduced the available time for cooking. It is important to note that the elaboration of homemade sourdough bread is a long process that requires time and dedication.

Bread is a basic food in the worldwide diet. Although white wheat bread, which is the most frequently sold bread, is usually manufactured without sourdough, it has good organoleptic and technological properties due to gluten proteins. By contrast, artisan bread is more expensive and oriented to specific demographics (and not the general public), although both profiles of consumers are starting to merge.

Actual food research in this field is mainly focused on the improvement of these products by using sourdough. Due to the nature of sourdough, the benefits and technological properties provided to the bakery products by these autochthonous microorganisms can be extended to all types of sourdough (including those made with GF flours). This capacity for improving the baked goods’ quality will depend on the microorganisms’ capacity to resist environmental stress, and to establish inter-dependent associations that will keep them stable along the entire fermentation process [52].

### 3.1. Factors Affecting Sourdough Microbiota

#### 3.1.1. Sourdough Fermentation Processes

It is fundamental to know the technological factors that affect and select the sourdough biodiversity, and those out of control, which can be responsible for the variability and dispersion observed in the results of different research articles in this field. Furthermore, it is important to be aware of manageable factors to optimize the process and focus this biotechnology into the final desired bakery product.

Among the non-controllable technological factors are the biochemical composition of the food ingredients (not only between flours from different grains, but also between the same flour type from different origins), and the house microbiota. It has been experimentally demonstrated that house microbiota is different depending on where the elaboration of the sourdough had been taking place (in a bakery, or in a relatively sterile environment, such as in a laboratory) [53].

On the other hand, some of the technological factors that can be controlled by the operator are:

1. Sourdough Type

**Figure 1:**
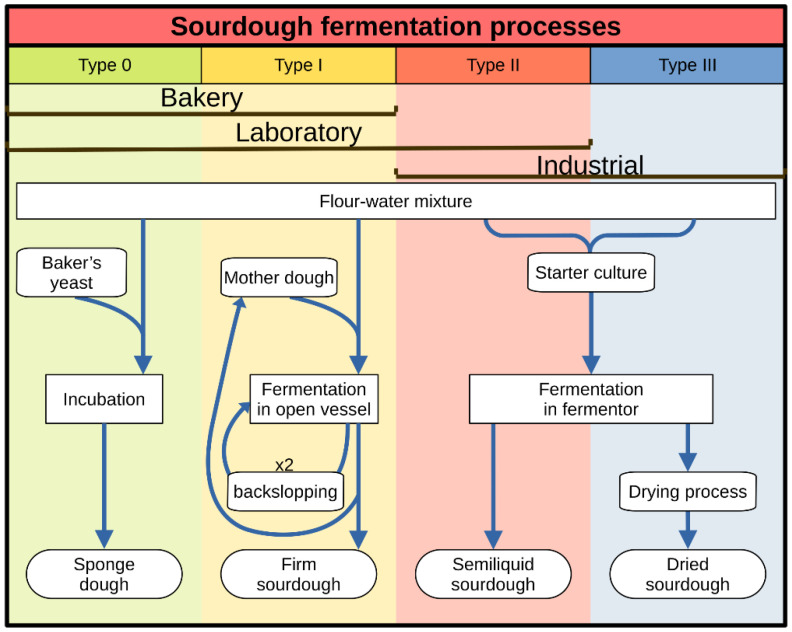
Types of sourdough fermentation processes according to the process technology applied. Adapted from: [54].

Depending on the process, four sourdough types can be distinguished (Figure 1) (some authors consider that, depending on certain parameters, there could also be subtypes) [54]:

- Type 0 sourdough is a type of pre-dough, also known as mother sponge, characterized by a short fermentation time at room temperature (RT, <30 °C). This provokes the initial propagation of native and exogenous LAB, with a higher proliferation rate compared to yeast, producing bioactive molecules and organic acids (lactic and acetic acids) that diminish the pH (pH~4). Given the short fermentation time, yeast growing is not enough in the sourdough and it is mandatory to add commercial yeast preparations. The microbiota that can be found in type 0 sourdough is a variety of LAB species; some of them are present in other types of sourdough, and others are not usually isolated and do not contribute to the improvement of the final product. It should be noted that in this type of sourdough there is no time to select those microorganisms with a higher adaptability to sourdough ecosystems, such as the yeast *Saccharomyces cerevisiae*. Typical examples are solid pre-ferments, such as *biga* from Italy and *pâte fermentêe* from France; and hydrated pre-ferments, such as the *levain levure* from north Europe, and *poolish* from Poland.
- Type I sourdough can be considered as the traditional sourdough, probably the one that spontaneously emerged in antiquity. Used in artisan bakeries and domestic settings, it considerably increases the quality of the final baked good. Type I sourdoughs have a long fermentation time at RT and are composed of very few microorganism species with the highest adaptation rates, the highest resistance, being the most competitive, and capable of stablishing solid associations between them. A typical example is the sourdough from San Francisco, mainly fermented by the LAB *Lactobacillus sanfranciscensis* (named because it was first isolated and described in this type of sourdough—reclassified as *Fructilactobacillus sanfranciscensis* [55]—) and the yeast *Candida humilis*. The association between these two microorganisms is very stable, since *Lb. sanfranciscensis* use maltose and *Candida humilis* use glucose, so they do not compete for the carbon source. They are also very competitive, displacing other species [56].
- Type II sourdough is a semiliquid fermented dough that can be bombed and used at an industrial scale. A starter culture is usually added to this type of sourdough, which is composed of LAB species that rapidly acidify the mixture and/or generate compounds that provide the aromas and flavors of traditional sourdough. Long fermentation times are used (two to five days) in only one step and at high temperatures (>30 °C). At these conditions, LAB rapidly proliferate (due to the high temperatures that facilitate their growing), with the consequent production of organic acids, the decrease of pH (pH < 3.4), and the yeast growing inhibition at this pH. This leads to the selection of acid-tolerant and thermophilic LAB (selection that is forced when commercial starter cultures are used) and requires adding industrial yeast. Some examples of *Lactobacillus* species isolated from type II sourdough are *Lactobacillus fermentum* (pro synonymon —pro synon.—*Limosilactobacillus fermentum)*, *Lactobacillus plantarum* (pro synon. *Lactiplantibacillus plantarum*) and *Lactobacillus reuteri* (pro synon. *Limosilactobacillus reuteri*) [55]; from rye sourdough, *Lb. amylovorus* is also frequently isolated [54].
- Type III sourdough is a freeze-dried type II sourdough to facilitate its commercialization and later industrial use.

2. Temperature of Fermentation

It has been described how the temperature of fermentation is a key factor for classifying the different types of sourdough, but inside the same type of sourdough, temperature is also a decisive factor; for example, the effect over the microbiota composition of a type I sourdough will not be the same if the RT is 20 °C or 35 °C.

The geographic location will determine the selection of the final microbiota. For example, *Lb. sanfranciscensis* (an endemic specie of type I sourdough) is not isolated in tropical climates, since it is a mesophilic species adapted to cold–temperate weathers. When the environmental temperature is high, it stimulates the proliferation of thermophilic species of *Lactobacillus*, such as *Lb. fermentum* (pro synon. *Limosilactobacillus fermentum*), *Lb. casei/paracasei* (pro synon. *Lacticaseibacillus casei*/*L. paracasei*) and *Lb. reuteri* (pro synon. *Limosilactobacillus reuteri*) [55,57].

3. Dough Yield

The dough yield (DY) is the proportion of water and flour in the sourdough. Low DY results in solid doughs, with higher acetic acid and lower lactic acid proportions, because of the inhibition of yeast by acetic acid. Indeed, the velocity of acidification of sourdough is also affected by DY, increasing both values proportionally: high DY results in a higher hydration of the dough and higher acidification velocity, probably due to a better diffusion of acids in a hydrated mixture [58].

4. Other Factors

Some other factors that can affect the sourdough elaboration process are [54,58]:

- The pH of the sourdough, affected by LAB or yeast presence and fermentation stage [58].
- Additional nutrient sources: traditional ingredients added to sourdough final mixes complement the nutrient content of the sourdough—e.g., adding mono- and disaccharides or different amino acid sources, thus affecting the intrinsic parameters for microbial growth [58,59] and the microbial composition itself [60].
- Ash content in the bran fraction of the flour. The bran fraction contains several minerals and micronutrients that can promote the growth of LAB in the sourdough. The ash content also influences the buffering capacity of the sourdough system that makes it possible to reach a higher total titratable activity [58].
- The amount of added salt can promote the presence of osmotolerant microorganisms such as yeast [54,58].
- The redox potential, depending on the oxygen availability, DY, frequency of dough refreshments, etc. [54,58].
- The resting time of the dough and its temperature; if it is performed at cold temperatures, it will favor microorganisms that are resistant to cold stress and to the absence of substratum [54].

#### 3.1.2. Instrumental Techniques for the Isolation and Identification of Microorganisms

Besides all variables that have been previously described, the instrumental techniques can provide new factors that have an impact on the results of the studies about sourdough microbiota; therefore, they should also be considered.

1. Sampling

Sampling is a critical step in all analytical techniques. As the whole sample cannot be analyzed, a representative aliquot must be selected, and the results extrapolated to the whole sample. Since the population of microorganisms varies along time and accordingly with the biotechnological process, the standardization of the sampling methodology, to obtain comparable results, is also required [61].

2. Fermentation Place

It should be considered that the microbiota analysis consists of the isolation and identification of the autochthonous microbiota, which comes not only from the food ingredients but also from the working place (e.g., the table and tools where the sourdough is made) and from the baker’s hands [62]. These environmental microorganisms are known as “house microbiota” [63].

If a microorganism is not present in some of the ingredients, and the sourdough is fermented in a relatively sterile environment (such as a laboratory), that microorganism will not be isolated from the sourdough. However, in highly contaminated environments (i.e., bakeries), with the presence of many different types of flour and other ingredients that can provide their own microbiota, it is reasonable to think that different microorganisms will be isolated in comparison to those found when the fermentation is produced in a laboratory [63].

Some authors have investigated whether the daily introduction of a type of flour in a bakery, and the fermentation of the corresponding type I sourdough, could define a house microbiota that could be used afterwards as an inoculum, similarly to the elaboration of wine, or cheese [62].

It has been hypothesized that house microbiota could mainly be responsible for isolating the same microorganisms from a specific sourdough produced in the same region. Nowadays, it has also been postulated that these similarities could also be due to the use of the same flour type, the same environmental conditions, and similar traditional food technological processes [64].

Furthermore, sourdoughs of every region and country are gaining importance as an identity sign, highlighting the need to preserve the biodiversity of each fermentation process. This is the reason why the non-profit initiative, Puratos Sourdough Library, a library of fermented doughs, was created in Belgium in 2013, to maintain sourdoughs worldwide. Currently, 1500 LAB species and 700 yeasts have been isolated from the 84 different sourdoughs collected by this library [65].

3. Isolation and Identification Techniques

During the last years, research about sourdough autochthonous microbiota has shown some variability in the obtained results. This lack of uniformity is mainly due to the different isolation and identification methodologies. Table 6 (Section 3.2.2.) specifies if the microorganism is identified by molecular techniques (based on genotypic factors), or by culturing methods (based on phenotype factors).

Phenotype methods are traditional identification methods of microorganisms, developed by culturing in agar plates. The sample is cultured in a non-selective enriched solid medium to isolate different colonies. Each colony is grown in liquid cultures that allow their rapid proliferation. They are then seeded again in specific and selective media for each type of microorganism. After confirming the isolation of single bacteria or yeast strains, its identity would be checked by different techniques, such as morphology assessment using microscopy methods, carbohydrate metabolism tests, or fermentation tests. With this methodology, it is necessary to know the type of microorganism we are searching for, since selective and differential growing media are used, with concrete substratum that allow the proliferation of only one, or a few species.

Genotype techniques are more recent and are based on molecular biology and the species identification by deoxyribonucleic acid (DNA). In this group, polymerase chain reaction (PCR and real-time PCR), microarray massive sequencing, and pyrosequencing techniques can be found [54,64].

### 3.2. Sourdough Autochthonous Microbiota

#### 3.2.1. Gluten-Containing Sourdough

Studies about the microbiota of GC sourdough are relatively recent. Spicher [66] and a Spanish research group headed by Benedito de Barber [67] were the first ones to investigate the autochthonous microbiota, with the intention of rescuing the sourdough tradition, as well as improving the quality of the mainly produced breads (based on short-time fermentations made by commercial yeast, with the only objective of producing CO_2_).

Numerous studies have been published investigating not only the beneficial properties that sourdough can provide to bakery products, but also which microorganisms (among all microbiota) are the responsible ones. Most of these studies are focused on wheat and, to a lesser extent, rye and barley.

The autochthonous microbiota of GC sourdough has been deeply studied during the last years. In a meta-analysis performed by Van Kerrebroeck et al. and published in 2017 [68], 583 sourdoughs were analyzed, and it was concluded that, in these sourdoughs, the most proliferating LAB were heterofermentative (which produce acetic acid, lactic acid, ethanol, and CO_2_ from the digestion of monosaccharides), although some homofermentative LAB (which only produce lactic acid) were also found. The isolated LAB species were mainly from the genera *Lactobacillus* [68]: *Lb. sanfranciscensis*, *Lb. plantarum*, *Lb.* *brevis* (pro synon. *Levilactobacillus brevis)* [55], *Pediococcus pentosaceus*, *L. paralimentarius*, and *L. fermentum* (LAB from the genera *Leuconostoc* and *Weisella* were also isolated, but in a lower proportion).

The main isolated yeast species were *S. cerevisae* (present in almost all bakeries, since it is used as a commercial yeast, and it is part of the house microbiota) and *C. humilis* (reclassified as *Kazachstania humilis*) [68]. In another review published in 2013, where 287 sourdoughs were analyzed, the main isolated yeasts were: *S. cerevisiae*, *C. humilis*, *Wickerhamomyces anomalus*, *Torulaspora delbrueckii*, *Kazachstania exigua*, *Pichia kudriavzevii*, and *Candida glabrata* [64].

#### 3.2.2. Gluten-Free Sourdough

**Figure 2:**
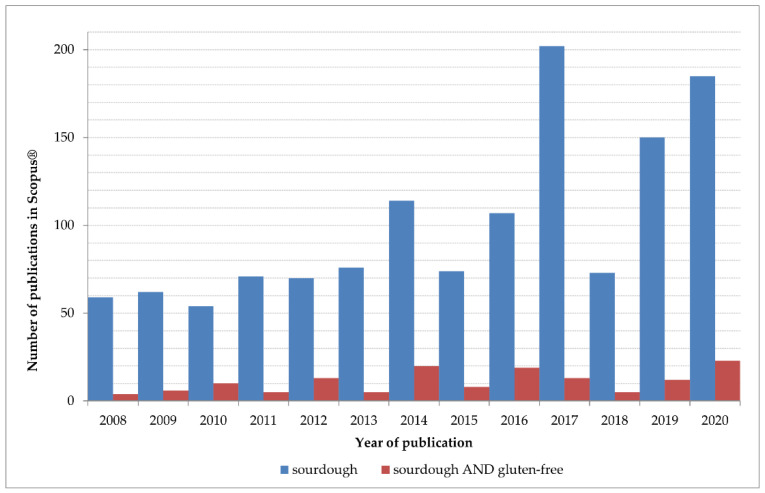
Number of publications retrieved from Scopus^®^ in the last 12 years using the terms “sourdough” or “sourdough AND gluten-free”.

Research about GF sourdough has not evolved in the same way than its GC counterpart. Figure 2 depicts a comparison of articles (published in Scopus during the last 12 years) by using the terms “sourdough”, or “sourdough AND gluten-free”. Before 2008, the search with “sourdough AND gluten-free” retrieves a scarce number of results, and before 2005, there are no results available in this database for these search terms.

The microorganisms (LAB and yeasts) isolated from different GF sourdough are presented in Table 6, according to information retrieved from different works and summarized by reviews from De Vuyst et al. [54] and Gobbetti et al. [59]. The sourdoughs are classified based on its origin (country), type of flour, fermentation method, fermentation place, and identification method. These results are difficult to compare, because of controllable and non-controllable factors that select the sourdough microbiota, including dough yield, propagation temperature, number and frequency of refreshments, use of starters, or the use of other ingredients.

**Table 6:** Microorganisms isolated from different GF sourdoughs.

Country ^1^	Flour Type ^1^	Propagation Method ^1^	Identification Method ^1^	Microorganisms Reported (LAB ^2^/Y ^3^)	Reference(s)
Argentina	Amaranth	Laboratory	Molecular	LAB: *Lactobacillus plantarum* ^4^	[59,69]
	Quinoa	Laboratory	Molecular	LAB: *Lb. brevis* ^5^, *Lb. plantarum*	[58,59]
		n.i.	n.i.	LAB: *Lb. plantarum*	[17,64]
Belgium	Teff	Bakery	Molecular	LAB: *L. brevis*, *L. helveticus*, *Lb. plantarum*, *L. sanfranciscensis*, *P. pentosaceus*	[70]
				Y: *K. exigua*	
		Laboratory	Molecular	LAB: *L. fermentum*, *Lb. plantarum*, *L. sanfranciscensis*, *W. cibaria*, and *P. pentosaceus*	
				Y: *S. cerevisiae*	
Botswana	Sorghum	n.i.	n.i.	LAB: *Lb. harbinensis* ^6^, *Lb. parabuchneri* ^7^, *Lb. plantarum*	[64,71]
China	Rice	Bakery	Molecular	LAB: *Enterococcus durans*, *E. faecium*, *Lb. plantarum*, *Pediococcus pentosaceus*	[59,72]
				Y: *Saccharomyces cerevisiae*, *Saccharomycopsis fibuligera*, *Torulaspora delbrueckii*, *Wickerhamomyces anomalus*	
	Maize	Bakery	Molecular	LAB: *E. durans*, *Lb. plantarum*, *P. pentosaceus*	
				Y: *S. cerevisiae*, *T. delbrueckii*, *W. anomalus*	
Ethiopia	Teff	Laboratory	Phenotypic	LAB: *E. faecalis*, *Lb. brevis*, *Lb. fermentum* ^8^, *Lb. plantarum*, *Leuconostoc mesenteroides*	[59,73]
		Laboratory	Molecular + phenotypic	LAB: *Lb. fermentum*, *Lb. graminis* ^9^, *Lb. parabuchneri*, *Lb. plantarum*	[59,74]
		Laboratory	Phenotypic	LAB: *E. casseliflavus*, *Lb. fermentum*, *Lactococcus piscium*, *Lc. plantarum*, *Lc. raffinolactis*, *Le. mesenteroides*, *P. acidilactici*, *P. pentosaceus*	[59,75]
				Y: *Candida humilis*, *C. tropicalis*, *Kazachstania exigua*, *Pichia norvegensis*, *S. cerevisiae*	
		Laboratory	Molecular + phenotypic	LAB: *Lb. fermentum*, *Lb. graminis*, *Lb. parabuchneri*, *Lb. plantarum*	[64,74]
France	Rice + buckwheat	Laboratory	Molecular	LAB: *Lb. sakei* ^10^	[59,76]
				Y: *C. humilis*	
Ghana	Maize	Bakery	Phenotypic	Y: *C. tropicalis*, *Kluyveromyces marxianus*, *P. kudriavzevii*, *S. cerevisiae*	[59,77]
Germany	Buckwheat	Laboratory	Molecular	LAB: *Lb. fermentum*, *Lb. helveticus*, *Lb. paralimentarius*, *Lb. plantarum*	[59,78]
				Y: not detected	
	Amaranth	Laboratory	Molecular	LAB: *Lb. paralimentarius*, *Lb. plantarum*, *Lb. sakei*, *P. pentosaceus*	[59,79]
		Laboratory, use of a starter including all LAB species on the right column	Molecular	LAB: *Lb. plantarum*, *Lb. sakei*, *P. pentosaceus*	
		Laboratory, use of a starter including all LAB species on the right column and *Lb. acetotolerans*, *Lb. brevis*, *Lb. casei*, *Lb. curvatus*, *Lb. sanfranciscensis*, *Lb. spicheri*, *Lc. lactis*, *Le. paramesenteroides* and yeast species *C. humilis*, *W. anomalus*, *P. kudriavzevii*, *S. cerevisiae*, *Torulaspora sp*	Molecular	LAB: *Lb. fermentum*, *Lb. helveticus*, *Lb. paralimentarius*, *Lb. plantarum*, *Lb. spicheri* ^11^	[59,78]
				Y: *C. glabrata*, *S. cerevisiae*	
		Laboratory	Molecular	LAB: *Lb. plantarum*, *Lb. sakei*	[64,79]
	Rice	Laboratory, use of a starter (mother sponge) including underlined species on the right column and *Lb. perolens*	Molecular + phenotypic	LAB: *Lb. paracasei*, *Lb. paralimentarius*, *Lb. spicheri*	[59,80]
				Y: *S. cerevisiae*	
		Laboratory, use of a starter including underlined species on the right column and yeast specie *P. membranifaciens*.	Molecular + phenotypic	LAB: *Lb. curvatus*, *Lb. fermentum*, * Lb. gallinarum*, *Lb. kimchii* ^12^, *Lb. plantarum*,* Lb. pontis* ^13^	
				Y: *P. kudriavzevii*, *S. cerevisiae*	
		Laboratory	Molecular	LAB: *Lb. fermentum*, *Lb. helveticus*, *Lb. plantarum*, *Lb. pontis*	[59,78]
				Y: *S. cerevisiae*	
		Laboratory	Molecular	LAB: *Lb. kimchii*, *Lb. paralimentarius*, *Lb. perolens* ^14^	[64,80]
	Maize	Laboratory, use of a starter including all species on the right column and *Lb. acetotolerans*, *Lb. brevis*, *Lb. casei*, *Lb. curvatus*, *Lb. sanfranciscensis*, *Lb. spicheri*, *Lc. lactis*, *Le. paramesenteroides* and yeast species *C. humilis*, *W. anomalus*, *Torulaspora sp.*	Molecular	LAB: *Lb. fermentum*, *Lb. helveticus*, *Lb. paralimentarius*, *Lb. pontis*	[59,78]
				Y: *P. kudriavzevii*, *S. cerevisiae*	
	Millet		Molecular	LAB: *Lb. fermentum*, *Lb. helveticus*, *Lb. pontis*	
				Y: *S. cerevisiae*	
	Quinoa		Molecular	LAB: *Lb. fermentum*, *Lb. helveticus*, *Lb. paralimentarius*, *Lb. plantarum*, *Lb. pontis*	
				Y: *P. kudriavzevii*, *S. cerevisiae*	
Italy	Quinoa	Laboratory	Molecular	LAB: *Lb. plantarum*	[17,59]
	Teff	Laboratory	Molecular	LAB: *Lb. plantarum*, *Lb. fermentum.*	[81]
				Y: *S. cerevisiae*	
Ireland	Buckwheat	Laboratory use of a starter use of a starter including all LAB species on the right column and *Lb. helveticus*, *Lb. paracasei*, *Lb. pontis*, *Lb. reuteri*, and yeast species *C. humilis* and *S. pastorianus*	Molecular	LAB: *Lb. amylovorus*, *Lb. brevis*, *Lb. fermentum*, *Lb*, *frumenti* ^15^, *Lb. paralimentarius*, *Lb. plantarum*, *Lb. sanfranciscensis* ^16^, *Leuconostoc argentinum* ^17^, *Weissella cibaria*	[59,82]
				Y: not detected	
		Laboratory	Molecular + phenotypic	LAB: *Lb. acidophilus*, *Lb. amylovorus*, *Lb. crispatus*, *Lb. fermentum*, *Lb. gallinarum*, *Lb. graminis*, *Lb. helveticus*, *Lb. plantarum*, *Lb.sakei*, *Lb. vaginalis*	[64,83]
		Laboratory	Molecular	LAB: *Lb. crispatus*, *Lb. fermentum*, *Lb. gallinarum*, *Lb. graminis*, *Lb. plantarum*, *Lb. sakei*, *Lb. vaginalis*, *Le. holzapfelii*, *P. pentosaceus*, *W. cibaria*	[59,83]
				Y: *K. barnetti*	
	Teff	Laboratory, use of a starter use of a starter including all LAB species on the right column and *Lb. helveticus*, *Lb. paracasei*, *Lb. pontis*, *Lb. reuteri*, and yeast species *C. humilis* and *S. pastorianus*	Molecular	LAB: *Lb. amylovorus*, *Lb. brevis*, *Lb. fermentum*, *Lb. frumenti*, *Lb. paralimentarius*, *Lb. plantarum*, *Lb. pontis*, *Lb. reuteri* ^18^, *Lb. sanfranciscensis*, *P. acidilactici*	
				Y: *K. barnettii*, *S. cerevisiae*	
		Laboratory	Molecular + phenotypic	LAB: *Lb. amylovorus*, *Lb. fermentum*, *Lb. gallinarum*, *Lb. plantarum*, *Lb. vaginalis* ^19^	[64,83]
		Laboratory	Molecular	LAB: *Lb. fermentum*, *Lb. gallinarum*, *Lb. pontis*, *Lb. vaginalis*, *Le. holzapfelii*, *P. pentosaceus*	[59,83]
				Y: *C. glabrata*, *S. cerevisiae*	
Morocco	Maize	n.i.	n.i.	LAB: *Lb. alimentarius*, *Lb. casei* ^20^	[64,84]
Nigeria	Maize	Laboratory	Molecular	LAB: *Lb. brevis*, *Lb. casei*, *Lb. fermentum*, *Lb. plantarum*, *Le. mesenteroides*, *P. acidilactici*	[59,85]
				Y: *C. albicans*, *S. cerevisiae*, *Schizosaccharomyces pombe*	
		Laboratory	Phenotypic	LAB: *Lb. brevis*, *Lb. casei*, *Lb. fermentum*, *P. acidilactici*, *P. pentosaceus*	[59,86]
		Laboratory	Molecular	LAB: *Lb. acidophilus*, *Lb. brevis*, *Lb. casei*, *Lb. fermentum*, *Lb. plantarum*	[64,85]
Portugal	Maize	Bakery	Phenotypic	LAB: *E. casseliflavus*, *E. durans*, *E. faecium*, *Lb. brevis*, *Lb. curvatus*, *Lc. lactis subsp. lactis*, *Leuconostoc* spp., *Streptococcus constellatus*, *S. equinus*	[59,64,87]
				Y: *S. cerevisiae*, *T. delbrueckii*, *W. anomalus*	
Saudi Arabia	Sorghum	Bakery	Phenotypic	LAB: *Lb. brevis*, *Lb. cellobiosus* ^21^, *Lb. lactis*, *P. pentosaceus*	[59,64,88]
				Y: *C. norvegensis*, *C. parapsilosis*, *Rhodotorula glutinis*	
Sudan	Sorghum	Laboratory	Phenotypic	LAB: *Lb. brevis*, *Lb. confusus* ^22^, *Lactobacillus* spp., *P. pentosaceus*	[59,89]
				Y: *C. intermedia*, *Debaromyces hansenni*	
		Laboratory	Phenotypic	LAB: *Lb. amylovorus*, *Lb. fermentum*, *Lb. reuteri*	[59,90]
				Y: *P. kudriavzevii*	
		Laboratory	Molecular + phenotypic	LAB: *E. faecalis*, *Lb. fermentum*, *Lb. helveticus*, *Lb. reuteri*, *Lb. vaginalis*, *Lc. lactis*	[59,64,90,91]

^1^ The sourdoughs are classified depending on the origin of the country, the type of flour, the propagation place (laboratory or bakery), and the identification method (molecular or phenotypic). Each row corresponds to an independent experiment. ^2^ LAB: lactic acid bacteria species; ^3^ Y: yeast species; n.i.: not indicated. ^4^ *Lactobacillus plantarum* (Orla-Jensen 1919) Bergey et al. 1923 pro synonymon (pro synon.) *Lactiplantibacillus plantarum* (Orla-Jensen 1919) Zheng et al. *2020*. ^5^ *Lactobacillus brevis* (Orla-Jensen 1919) Bergey et al. 1934 pro synon. *Levilactobacillus brevis* (Orla-Jensen 1919) Zheng et al. 2020. ^6^ *Lactobacillus harbinensis* Miyamoto et al. 2006 pro synon. *Schleiferilactobacillus harbinensis* (Miyamoto et al. 2006) Zheng et al. 2020. ^7^ *Lactobacillus parabuchneri* pro synon. *Lentilactobacillus parabuchneri* (Farrow et al. 1989) Zheng et al. 2020. ^8^ *Lactobacillus fermentum* Beijerinck 1901 pro synon. *Limosilactobacillus fermentum* (Beijerinck 1901) Zheng et al. 2020. ^9^ *Lactobacillus graminis* Beck et al. 1989 pro synon. *Latilactobacillus graminis* (Beck et al. 1989) Zheng et al. 2020. ^10^ *Lactobacillus sakei* Katagiri et al. 1934 pro synon. *Latilactobacillus sakei* (Katagiri et al. 1934) Zheng et al. 2020. ^11^ *Lactobacillus spicheri* Meroth et al. 2004 pro synon. *Levilactobacillus spicheri* (Meroth et al. 2004) Zheng et al. 2020. ^12^ *Lactobacillus kimchii* Yoon et al. 2000 pro synon. *Companilactobacillus kimchii* (Yoon et al. 2000) Zheng et al. 2020. ^13^ *Lactobacillus pontis* Vogel et al. 1994 pro synon. *Limosilactobacillus pontis* (Vogel et al. 1994) Zheng et al. 2020. ^14^ *Lactobacillus perolens* Back et al. 2000 pro synon. *Schleiferilactobacillus perolens* (Back et al. 2000) Zheng et al. 2020. ^15^ *Lactobacillus frumenti* Müller et al. 2000 pro synon. *Limosilactobacillus frumenti* (Müller et al. 2000) Zheng et al. 2020. ^16^ *Lactobacillus sanfranciscensis* corrig. (ex Kline and Sugihara 1971) Weiss and Schillinger 1984 pro synon. *Fructilactobacillus sanfranciscensis* (Weiss and Schillinger 1984) Zheng et al. 2020. ^17^ *Leuconostoc argentinum* Dicks et al. 1993 pro synon. *Leuconostoc lactis* Garvie 1960. ^18^ *Lactobacillus reuteri* Kandler et al. 1982 pro synon. *Limosilactobacillus reuteri* (Kandler et al. 1982) Zheng et al. 2020. ^19^ *Lactobacillus vaginalis* Embley et al. 1989 pro synon. *Limosilactobacillus vaginalis* (Embley et al. 1989) Zheng et al. 2020. ^20^ *Lactobacillus casei* (Orla-Jensen 1916) Hansen and Lessel 1971 pro synon. *Lacticaseibacillus casei* (Orla-Jensen 1916) Zheng et al. 2020. ^21^ *Lactobacillus cellobiosus* (Rogosa et al. 1953) pro synon. *Limosilactobacillus fermentum* (Beijerinck 1901) Zheng et al. 2020. ^22^ *Lactobacillus confusus* (Holzapfel and Kandler 1969) Sharpe et al. 1972 pro synon. *Weissella confusa* corrig. (Holzapfel and Kandler 1969) Collins et al. 1994.

The type of sourdough determines the microorganisms that will proliferate. Studies included in both reviews [54,59] are mainly focused on type 0 and type I sourdoughs, the most interesting ones.

Selecting the same type of sourdough (made from corn), Vogelmann et al. ([84] included in the review published by Luc De Vuyst et al. in 2017 [54]) isolated different species when it was fermented in Germany, or in China, with the only exception of *S. cerevisiae*. Considering that type I sourdough is fermented at RT, this value fluctuates between countries, and could be a main determinant for the selection of microorganisms. Besides that, the corn sourdough from China used a traditional starter culture, named *Jiaozi*, which could have addressed the selection of the final microbiota composition [92].

**Figure 3:**
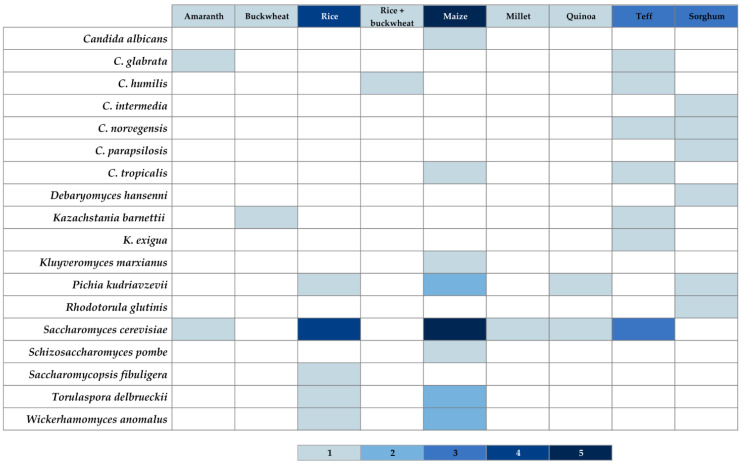
Heat map for yeast species. The presence of certain yeast species, isolated from the specific GF sourdough indicated in table header is described with colored cells. The intensity of blue color, as shown in the scale at the bottom, represents the least (1) and the most (5) frequent isolations, within the findings of this review. The intensity of blue color in the table header cells represents the least (1) and the most (5) analyzed type of GF sourdough within the examined results. Authors’ own elaboration based on the findings of the present review.

**Figure 4:**
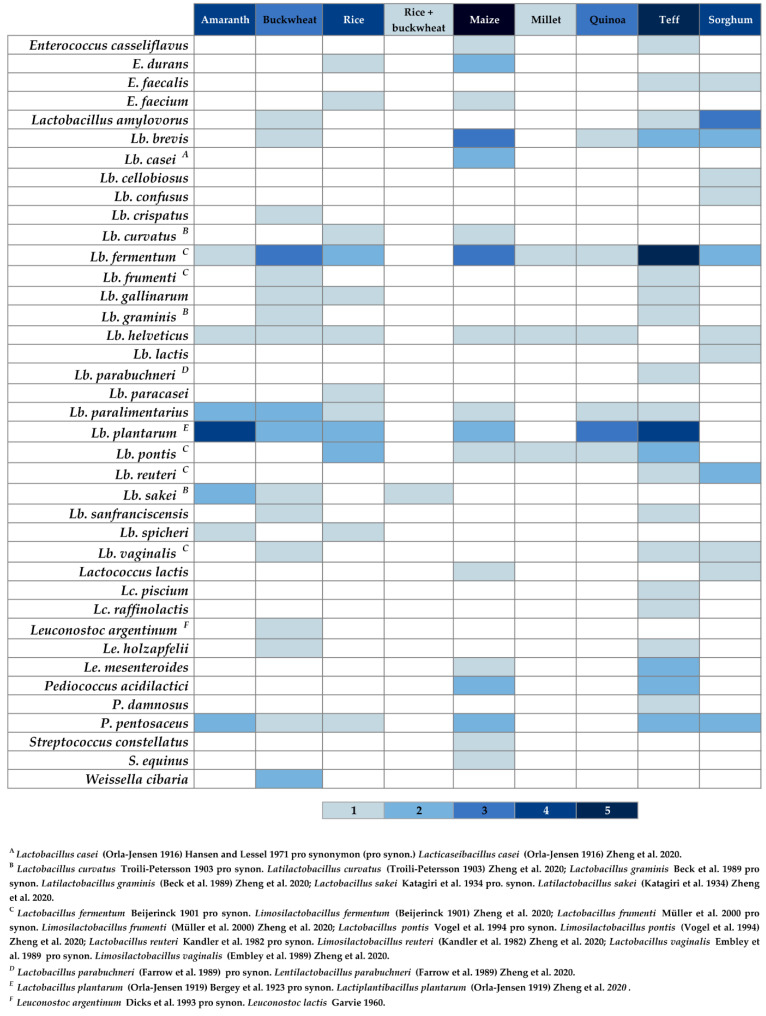
Heat map for LAB species. The presence of certain LAB species, isolated from the specific GF sourdough indicated in table header, is described with colored cells. The intensity of blue color, as shown in the scale at the bottom, represents the least (1) and most (5) frequent isolations, within the findings of this review. The intensity of blue color in the table header cells represents the least (1) and most (5) analyzed type of GF sourdough within the examined results. The bacterial nomenclature was revised according to Zheng et al. [55] and Parte et al. [85]. Authors’ own elaboration based on the findings of the present review.

Figure 3 and Figure 4 show heat maps depicting the frequency of isolation of different yeast and LAB species from different GF sourdoughs, based on the findings of the present review.

A similar scenario than the one described for GC sourdoughs is observed in Figure 3, where the frequencies of different yeast species are shown. *S. cerevisiae*, being used as a commercial starter culture, is part of the bakery’s environment and can be isolated from most of the GF sourdoughs. If we compare these results with the ones presented in Table 6, the absence of *S. cerevisiae* in the sourdoughs is related to a fermentation performed in the laboratory.

In Figure 4, where the frequencies of different LAB species are shown, there are some recurrent bacteria that can be widely isolated due to their colonization ability. For example, *Lb. fermentum* has been isolated from practically all sourdoughs, indicating that this microorganism should be specially considered in sourdough biotechnology. The following ones, in decreasing order of frequency, are *Lb. plantarum* and *P. pentosaceus*. However, *Lb. sanfranciscensis*, considered as an endemic bacteria of type I GC sourdoughs, has only been isolated in two types of GF flours (buckwheat and teff), and not in all cases.

According to the Spanish bread quality standard [86], it can be indicated that a bread is made with sourdough as long as it is in a proportion equal or superior to 5% of the total weight of the flour of the final dough. The most-used proportion of gluten-free sourdough is usually around 20% [34,87], since it seems to give better results. However, it has been observed that this amount depends on the type of flour used to make the gluten-free sourdough. For example, in the elaboration of GF bread with sourdough from chestnut flour, good results were observed with concentrations between 30 and 50% [88]. Using both fresh and freeze-dried rice sourdough flour, the best sensory results were obtained with 10 to 20% of added sourdough [89]. In another work, the best results were obtained using amounts of 20 to 30% with both fresh and freeze-dried sourdough from buckwheat flour [90]. In a similar research using both fresh and freeze-dried sourdough from amaranth, the best sensory results were obtained with an amount of 10%, GF bread being sensorially rejected if the concentrations added were of 20% [91].

## 4. Identification of Microorganisms Capable of Producing Hydrocolloid-Like Compounds

The overall benefits that sourdough provides to bakery products have already been described: improvements at organoleptic (taste, texture, and aroma) and nutritional (hydrolysis of anti-nutrients, such as phytic acid) levels, the extension of shelf life, and synthesis of functional molecules (prebiotics, antioxidants, antifungals, peptidases that degrade immunogenic peptides, etc.).

All these properties are mainly attributable to the microbiota (bacteria and yeasts) that proliferates and is established in the sourdough. As a result of the metabolic processes, these microorganisms synthesize and release molecules with diverse properties and functionalities. Within this biodiversity, bacterial contributions are the most relevant. The main function of yeasts is the CO_2_ production, although they also contribute to the synthesis of metabolites, such as alcohols and derived esters, and the characteristic flavor and aromas of the crumb of fermented products [93].

Analyzing the published literature, it has been observed that bacteria are the microorganisms that contribute most to these technological improvements by synthesizing a diverse group of molecules, called EPS. These molecules are long-chain carbohydrates (polysaccharides) that widely differ among them in terms of their molecular characteristics, composition, structure, and even mechanisms by which they are synthesized [94,95,96]. In sourdough, EPS can improve technological properties, avoiding the addition of other hydrocolloids. Moreover, they can present other properties, such as prebiotic, immunomodulatory, antioxidant, pathogen inhibition, etc. [97,98,99].

There are two types of EPS—heteropolysaccharides (HePS) and homopolysaccharides (HoPS) [96,99,100,101,102,103]:

- HePS are described as such because the sugar polymer chain is made of different monosaccharides, usually D-galactose, D-glucose, R-rhamnose and, to a lesser extent, other N-acetylated monosaccharides, varying from two to eight different monomers, and with a molecular weight up to 10^6^ Da. A large variety of HePS can be synthesized by LAB, depending on the type of monosaccharides, bonds between these monosaccharides, and spatial configurations (linear vs. branched). As an example, Suzuki et al. studied how *Lactococcus lactis* can synthesize a high number of different HePS [102]. HePS are synthesized from sugar–nucleotide precursors, intracellularly (in the cytoplasm), and in small quantities, usually between 10 and 166 mg/L. The yield of this synthesis depends on several factors: by optimizing some culture parameters of *Lb. plantarum*, Ismail and Nampoothiri achieved a final EPS concentration of 1.2 g/L [103]. Xanthan and gellan gums are HePS synthesized by bacteria belonging to phylum “*Proteobacteria”*.
- HoPS are polymers based on a single type of monosaccharide (glucose or fructose), and, because of this, they are recognized as glucans or fructans (also designated as fructooligosaccharides or FOS) [96,100]. Its synthesis is extracellular, from sucrose, by the action of enzymes (glycosyl hydroxylases), and with a molecular weight greater than HePS (>10^6^ Da). For the polymerization of glucose or fructose, these enzymes employ the energy of the glycosidic bond. HoPS are synthesized by different genera of LAB (mainly, *Lactobacillus*, *Streptococcus*, *Leuconostoc*, *Oenococcus* and *Weissella*) and in an amount greater than HePS, reaching up to 10 g/L. In addition to this first classification of HoPS (in glucans and fructans), these compounds are also classified based on the carbons involved in the glycosidic linkages of the backbone chain of the polymer.
  ○ Within the group of glucans, the following types are recognized: *dextrans*, *mutans*, *reuterans*, and *alternans*. Dextrans are the HoPS with the most technological relevance, being the only EPS synthesized at an industrial level, widely used as, for example, a thickener for jams and ice cream: they reduce crystallization, increase moisture retention, and do not affect taste.
  ○ Two types of fructans can be distinguished: *inulin* and *levan*. As its prebiotic properties, inulin is acquiring a greater role in the current market. Recently, it has been reported that fructans can induce gastrointestinal symptoms in individuals with self-reported non-celiac gluten sensitivity [104].

Normally, LAB species that synthesize HoPS only produce a single glycosyl hydroxylase enzyme and, consequently, a single type of EPS. There are some exceptions, such as *Leuconostoc mesenteroides*, which produces dextran, alternan and levan [96].

Once the EPS types are exposed, and which LAB are related to their synthesis have been identified, the next step will be the study and physical–chemical characterization of each EPS, to determine its activity and technological properties which are contributing or could contribute to the doughs [100]. From the point of view of the GF bakery industry, the most important property of certain EPS is to aid to resemble texture and appearance of GF baked goods to wheat-based baked products.

At this point, it is essential to remember that because EPS are a very heterogeneous group of compounds, not all of them have the same properties; therefore, not all of them can emulate the functions of gluten molecules in doughs.

Current research is focused on the study of each type of EPS and on the identification of those with technological potential as substitutes of gluten. This will allow three approaches, based on sourdough and LAB, to try to solve the problem of low sensory quality of gluten-free products [21,81,105,106]:

- Using mixtures of GF flours, where each flour supplies a type of bacteria that produces the EPS that we are looking for.
- Using controlled fermentation processes oriented to the development of the microbiota of interest.
- Using commercial starters based on bacteria strains selected because of their technological potential.

The technological and functional properties of EPS is due to its ability to act as hydrocolloids in the dough [58,100]: (i) increasing water absorption, (ii) improving rheology, (iii) increasing the final volume, (iv) increasing the softness of the crumb, and (v) increasing the shelf life by avoiding starch retrogradation.

We have already seen that in the GF products’ industry, the use of hydrocolloids is widely employed, HPMC and xanthan gum (which is the only microbial EPS with relevance as an additive) being the most widely used [101]. The characterization of certain EPS confirms that, in the dough, they behave in a similar way to these exogenous additives. They are also capable of interacting with water molecules and forming a mesh-like structure with gel properties, which increases CO_2_ retention (although the exact mechanisms of this behavior are still unknown) [101].

The EPS that are most used for this purpose are the HoPS because they are synthesized extracellularly, reaching higher concentrations that are relevant at a functional level. It is estimated that the amount of HoPS synthesized can reach values around 0.8% *w*/*v*, and considering that hydrocolloids are usually added in dough at 0.3% *w*/*v*, it is logical to think that they could be used as potential substitutes of these additives [21,101].

Zannini et al. presented a brief classification of HoPS, the corresponding LAB that are involved in their synthesis and the main food industrial applications of HoPS in an interesting mini-review [96]. The EPS synthesized by different LAB, and the properties attributed to them in experimental tests on specific sourdoughs has also been reviewed by Lynch et al. [101].

The conditions of EPS production by sourdough lactobacilli depend on several factors, such as sourdough composition (available carbon sources, mainly sugars, and their concentration, nitrogen sources, content of other nutrients), fermentation conditions (time, temperature, oxygen, pH), *Lactobacillus* species, and the type of flour used, among others [100,101,107,108,109]. The concentration of fermentable sugars present in the dough affects the EPS microbial synthesis [110]. Sucrose concentration is of particular relevance for some species, such as *Weissella cibaria* [96,110,111].

Considering this information, we could think that it is as simple as selecting some LAB and designing a starter culture with technological properties. This selection would be made based on its ability to synthesize EPS and other properties of interest, such as its growth kinetics, its acidification capacity, its fermentation quotient (ratio between acetic acid and lactic acid), its release of amino acids involved in the formation of aroma and flavor, or its ability to hydrolyze immunogenic gluten peptides (eliminating possible cross contamination and making safer products for CD patients) [111].

However, considering what a sourdough is, the inherent complexity and the variability factors that affect this ecosystem, it is logical to think that the development of these starters is somewhat more complex.

Experimental tests suggest that the selection of these LAB should be carried out on the endemic bacteria of each sourdough; that is, they should be isolated in that specific process, in such a way that we can ensure that they will be adapted to that substrate and fermentation conditions and be competitive enough to outperform the rest of the present microorganisms [112].

Again, we find that research on GF sourdoughs is scarce, and the use of commercial starters tested (with good results) in GC doughs is not useful in GF flours. Moroni et al. investigated two commercial starters for GC doughs in buckwheat and teff flours, with negative results. In fact, both *Lb. helveticus* as *Lb. paracasei*, which were both part of this starter, were not isolated from the mature sourdoughs [77]. Galle et al., using *Lb. buchneri* (producer of HePS) in sorghum sourdough, also obtained loaves with a loss of elasticity with respect to the control, a phenomenon that did not occur in doughs made with wheat [113].

Therefore, it is important to select bacteria strains within the native microbiota with desirable properties that allow rapid adaptation, intense acidification, and a positive influence at both a technological and nutritional level [114].

As some examples of positive experimental results, Galle et al. showed that sorghum sourdoughs were improved with the addition of *W. cibaria* and *Lb. reuteri* by producing dextran and fructan, respectively [105]. Wolter et al. also optimized the use of *W. cibaria* in their bread model made with buckwheat, quinoa, sorghum, and teff flours. They also verified how the type of flour influenced the amount of dextran synthesized by this bacterium [87]. In a research study developed by Nami et al., the use of sourdoughs with starters based on combinations of four LAB species improved the quality and shelf-life of GF pearl millet bread, with starters based on *L. brevis* and *L. paralimentarius* being the most successful ones [106]. Dingeo et al. achieved good nutritional values in gluten-free muffins baked with a teff Type-I sourdough, dominated mainly by *Lb. plantarum*, *Lb. fermentum* and *S. cerevisiae* [76]. The interpretation we can give is that further investigation is necessary for each particular case. Starting from bacteria present in the sourdoughs of each type of flour and specific process, those most interesting (from a technological point of view), could be selected.

On the other hand, the use of starters provides additional benefits to the use of sourdoughs since it directs the selection of microorganisms in some way [57]. In addition, it can be very useful in type II sourdoughs, so that not only acidification occurs, but also benefits attributable to the use of sourdough.

## 5. Concluding Remarks and Future Perspectives

Once the main functional and technological properties of the most commonly used additives and adjuvants in GF bakery have been described, the reviews selected to develop Section 2 of this paper are presented in Table 2, Table 3, Table 4 and Table 5. The descriptors depicted in these tables are: (i) the type of flour used in the preparation; (ii) the additive or mixture of additives, and their concentration (if it was mentioned in the article); and (iii) both the positive and negative technological properties described in the final product. Most of the studies refer to GF bread and, in almost all cases, the type or types of flours used in the preparation are also indicated (when the study refers to another type of product, it is also indicated in the tables). The overall conclusion of Section 2 is that it is complicated to establish beneficial or harmful properties (from a technological point of view), of any additive, since they are based on a set of variables (e.g., food matrix, type of additive, concentration at which it will be used, or interactions between the different ingredients and the subsequent processing). As with any other ingredient, additives make the final product more expensive, and need to be tested for every specific condition, since their technological contribution depends on the characteristics of each dough. In addition, additives must be declared on the label, which is a problem for some consumers who are reluctant to use food additives.

From Section 3, it can be concluded that there is a high variability of microorganisms present in GF sourdough. The papers analyzed suggest that, similarly to GC flours, their GF counterparts have endemic LAB that can be isolated in practically all GF sourdoughs. Therefore, the study of autochthonous microbiota highlights that there are some species strong enough and adapted to the ecosystem that can be considered as endemic in these sourdoughs, and able to compete and proliferate independently of the process. However, more studies are needed to compare the results and to correctly identify autochthonous microbiota in GF sourdough.

It can be postulated from Section 4 that each sourdough contains at least one EPS-producing *Lactobacillus* strain, so the use of fermentation could replace additives as functional ingredients. From the knowledge of the microbiota present in the GF sourdoughs and the EPS synthesized by these microorganisms, the best species could be selected (based on their technological and nutritional potential) as starter cultures. These starters, formed by bacteria and yeasts selected for their technological characteristics, could improve bakery processes (including products fermented at industrial level). Further research is necessary in this field to develop the full potential of an economic and ecological biotechnology, such as the use of sourdough, which is capable of positively influencing all the parameters with which we measure the final quality of GF products.

## Figures and Tables

**Table 1:** Properties of sourdough responsible of improving the quality of bakery products.

Sourdough Property/Function	References
Sensory improvements	[17]
Nutritional improvements	[18,19]
Functional improvements	[20]
Shelf-life extension	[21]

**Table 2:** Hydrocolloids used in gluten-free baked goods.

Food Product	Cereal(s) or Pseudo-Cereal(s) Used in the Product	Main Flour(s)	Hydrocolloids	Technological Outcome	Reference(s)
GF bread	Brown rice	Brown rice	Xanthan gum, guar gum, xanthan-locust bean gum, MC ^1^, CMC ^2^, HPMC ^3^	↑^4^ Porosity, ↑ cohesiveness and elasticity	[25,26]
GF bread	Buckwheat	Buckwheat flour	0.14% xanthan gum	↑ Bread volume ↓^5^ Crumb hardness/firmness	[12,27]
GF bread	Buckwheat	Buckwheat flour	Guar gum, HPMC, tragacanth gum	↑ Crumb alveoli resistance, ↑ elasticity	[25]
GF ^7^ bread	Teff, buckwheat, rice maize	Teff, buckwheat, rice, or maize flours	1.5% HPMC	dov ^6^	[12]
	Rice	Rice flour and potato starch	Fructans (such as inulin)	↑ Bread volume ↓ Crumb hardness	[28]
	Maize	Maize starch, potato starch	Inulin (<10 polymerization degrees)	↑ Bread volume ↓ Crumb hardness	
	Maize	Maize starch, zein	HPMC, high β-glucan oat bran	Positive rheology, good crumb structure	
	Maize	Maize starch, potato starch	5% Inulin	↑ Bread volume (4%) ↓ Crumb hardness	[29]
	Maize	Maize starch, potato starch	8% Inulin	↑ Bread volume (9%) ↓ Crumb hardness Wrinkling of the crust	
	Rice	Rice flour and potato starch	4% to 12% ITFs ^8^ (Raftilose^®^ Synergy1)	↑ Specific volume, darker crust, appealing crust and crumb	
	Rice	Rice flour	Inulin	↑ Volume, delayed staling, improved crumb, smoother crust	
	Rice	Rice flour, potato starch, cassava starch, sour tapioca flour	ITFs (inulin, FOS ^9^)	Color and porosity improvements Improved texture, taste and flavor	
	Maize, rice	Maize flour, rice flour, inactive soy flour	CMC or xanthan gum	dov	[30,31]
	Rice	Maize flour, carob flour, resistant starch (RS)	Carob flour, resistant starch (RS)	Low crumb firmness and improved porosity values with 15 g carob flour, 10 g RS, 10 g protein and 140 g water/100 g flour	
	Maize	Maize starch, potato starch	Flaxseed mucilage	Improved sensory acceptance	[15]
	Amaranth	Maize starch, amaranth flour, pea isolate	Psyllium	Improved final product quality	
	Rice, quinoa	Rice flour, quinoa flour	Xanthan gum	dov	[32]
	Rice, buckwheat	Rice flour, buckwheat flour			
GF layer cakes	Rice	Rice flour	Inulin, oat fibers, guar gum	Same volume as control ↑ Crumb firmness ↓ Elasticity	[28]
GF cheese bread	Maize	Pre-cooked cornflour, cassava starch	9% FOS	↓ Hydration; ↑ solubility of starch–FOS mixtures	[29]
GF bread	Maize	Maize flour	1.77% HPMC	↑ Bread volume ↓ Crumb hardness/firmness	[12]
“Empanadas” and piecrusts	Maize	Maize starch	Guar gum, HPMC, xanthan gum	↑ Elasticity	[25]
GF bread	Maize	Maize flour, maize starch	Xanthan gum	↑ Specific volume ↓ Crumb hardness	
GF bread	Maize	Maize starch, potato starch	Pectin, whey protein	dov	[32]
GF bread	Maize	Maize flour, maize starch	Guar gum, pectin	↓ Firmness, ↓ crumb hardening	[25]
GF bread	Rice	Rice flour	2.2% HPMC	dov	[12]
GF bread	Rice	Rice flour	HPMC	↑ Elasticity and viscosity	[25]
GF bread	Rice	Rice flour	HPMC	dov	
GF bread	Rice	Rice flour	Xanthan gum, carob gum, guar gum, HPMC	↑ Viscoelasticity	
GF bread	Rice	Rice flour	HPMC	↑ Specific volume	
GF bread	Rice	Rice flour	HPMC, xanthan gum	↑ Specific volume	
GF bread	Rice	Rice flour	HPMC	↓ Crumb firmness	
GF bread	Rice	Rice flour	HPMC	↑ Moisture content Enhanced sensory properties	
GF bread	Rice	Rice flour	HPMC, guar gum, CMC	↑ Specific volume	[25]
GF flat bread	Rice	Rice flour	15 g/kg xanthan gum 10 g/kg CMC 10 g/kg xanthan gum	↑ Crumb alveoli size ↑ Crumb porosity ↑ Dough yield	[15]
GF bread	Rice	Rice flour	HPMC	dov	[32]
GF bread	Rice	Rice flour	HPMC, β-glucan	dov	
GF bread	Rice	Rice flour	Xanthan gum, guar gum, carob gum	dov	
GF cake and muffin products	Rice	Rice flour	Tragacanth gum, xanthan gum	dov	
GF bread	Rice	Rice flour, carob flour, resistant starch	Carob gum, DATEM^®^, whey protein concentrate, α-amylase, transglutaminase, hemicellulase	dov	
GF bread	Rice, buckwheat	Rice flour, buckwheat flour	Xanthan gum	dov	
GF bread	Rice, maize	Rice flour, maize flour, soy flour	Carrageenan, alginate, xanthan gum, CMC	↑ Consistency, ↑ starch retrogradation, ↑ amylopectin retrogradation	[25]
GF bread	Rice, maize	Rice flour, maize starch	Xanthan gum	dov	
			CMC, pectin, agarose, xanthan gum	↑ Elasticity ↑ Dough strength	
			CMC, xanthan gum	↓ Crumb firmness ↑ Crumb porosity	
Egyptian balady flat bread	Rice, maize	Rice flour, maize starch, potato starch	Xanthan gum, guar gum	↓ Loss of moisture ↓ Hardness/firmness	
GF bread	Brown rice, maize, buckwheat	Brown rice flour, maize starch, soybean flour, buckwheat flour	Xanthan gum, Konjac gum	↓ Elasticity, cohesiveness, and resilience	
	Rice, maize	Rice flour, maize starch, chestnut flour	HPMC, lupine protein, vegetable fiber; guar gum, skimmed milk, cellulose	dov	[32]
	Rice, maize	Rice flour and maize starch	HPMC, skimmed milk, egg powder, soy protein, xanthan gum	dov	
			HPMC, vegetable fiber (bamboo, oat, pea, potato)	dov	
GF bread	Rice, maize	Rice, maize, maize starch	Xanthan gum	↑ Color improvements, ↑ Volume, hydration	[25]
GF bread	Rice, maize, quinoa	Rice flour, maize flour, maize starch, quinoa flour	HPMC, amyloglucosidase, α-amylase	↑ Volume, ↑ firmness	[32]
GF bread	Rice, maize, rice	Rice flour, maize flour, rice starch, rice protein	HPMC, carob gum, guar gum, psyllium, beetroot fiber, amylase	dov	
GF bread	Sorghum, maize	Decorticated sorghum flour, maize starch	Xanthan gum	dov	[25]
GF bread	Teff	Teff flour	0.04% xanthan gum 2% HPMC	↑ Bread volume ↓ Crumb hardness/firmness	[12]

^1^ MC: methylcellulose; ^2^ CMC: carboxymethylcellulose; ^3^ HPMC: hydroxypropyl methylcellulose; ^4^ ↑: results in an increase of the mentioned feature; ^5^ ↓: results in a decrease of the mentioned feature; ^6^ dov: dependent on variables; ^7^ GF: gluten-free; ^8^ ITFs: inulin-type fructans; ^9^ FOS: fructooligosaccharides.

**Table 3:** Proteins used in gluten-free baked goods.

Food Product	Cereal(s) or Pseudo-Cereal(s) Used in the Product	Main Flour(s) Used in the Product	Protein Supplementation/Additives	Technological Outcome	Reference(s)
GF ^1^ bread	Rice	Rice flour, cassava starch, soy flour	7.5% soy 7.8% milk powder	↑ ^2^ Nutritive value, without sensorial changes	[12]
	Rice	Rice flour	Bovine plasma protein	dov ^3^	[30]
	Rice	Rice flour	Bovine serum albumin	dov	
	Maize	Maize starch, potato starch	Collagen	dov	
	Variable	Variable	Egg, caseinate, whey protein, milk protein	dov	[30,41]
	Variable	Variable	Egg	Improved structure, stable foaming, and gas retention	[12]
	Variable	Variable	Lactose free milk powder	dov, darkening of the crumb	
Precooked rice pasta	Rice	Rice flour, yellow pea flour, chickpea flour, lentil flour	Legume protein	Increased protein and dietary fiber content	[15]
GF bread	Rice, buckwheat, quinoa	Rice flour, quinoa flour, buckwheat flour, potato starch	Quinoa protein	↑ Elasticity and dietary fiber content improved dough structure	
GF bread	Rice	Rice flour	Rice bran protein concentrate	↑ Elasticity, shear strength, volume, gas retention and shelf life	
GF bread	-	Soy flour	Soy	dov (↑ loaf volume, ↓ ^4^ crumb hardness)	[12,42,43]
GF bread	-	Soy flour	Soy protein	dov	
GF bread	Variable	Soy flour	Soy protein, milk powder	dov	
GF bread	Variable	Soy flour	Soy, pea	dov	
GF bread	Variable	Starch from different sources	Whey protein	dov	[12,30]
GF bread	Maize	Maize flour	Zein	dov	[12,30]
Egg yolk muffins	Maize	Maize	Egg yolk granulates, apple pectins, gelatine	dov	[32]
GF bread	Maize	Maize starch, carob germ flour	Carob protein, HPMC ^5^	dov	[12]
GF doughs	Maize	Unmodified maize starch	Zein, HPMC	dov	
GF muffins	Maize	Maize starch, kidney bean flour, field pea flour, amaranth flour	Protein isolates	dov	[32,41,44]
GF muffins	Rice		Egg, fructose, inulin, sucralose	dov	
	Rice		Jambolan fruit pulp, soy Protein isolates, glycerol monostearate, xanthan gum	dov	
	Rice		Soya bean protein isolate, pea protein isolate, egg white isolate, casein, xanthan gum	dov	
	Rice		Soy protein isolates, glycerol monostearate, xanthan gum, black carrot dietary fiber concentrate	dov	
GF bread	Buckwheat, rice	Buckwheat flour, rice flour, chickpea flour	Green mussel protein hydrolysates	dov	
GF bread	Wheat	Wheat starch	6% whey protein	Darker crust, white crumb, ↑ volume, improved texture	[12]
	Wheat	Wheat starch	Whey protein	dov	

^1^ GF: gluten-free; ^2^ ↑: results in an increase of the mentioned feature; ^3^ dov.: dependent on variables; ^4^ ↓: results in a decrease of the mentioned feature; ^5^ HPMC: hydroxypropylmethylcellulose.

**Table 4:** Enzymes used in gluten-free baked goods.

Food Product	Cereal(s) or Pseudo-Cereal(s) Used in the Product	Main Flour(s)	Enzymes/Additives	Technological Outcome	Reference(s)
GF **^1^** bread	Brown rice, buckwheat, maize, oat sorghum or teff	Brown rice, buckwheat, maize, oat, sorghum or teff flours	0.1 or 10 U ^2^ of TGase ^3^/g of protein	Depending on protein source and enzyme dosage	[12,47]
GF bread	Buckwheat, brown rice	Buckwheat flour, brown rice flour	0.1 to 10 U of TGase/g protein	↑ ^4^ Increased batter pseudoplasticity, ↑ water holding capacity, improved crumb texture and structure	[15]
GF bread	Buckwheat, sorghum, or maize	Buckwheat, sorghum, or maize flours	0.01% or 0.1% proteases	Liquid-like batters, poor viscoelastic behavior, ↓ ^5^ gas-holding capacity	[12]
GF bread	Buckwheat, rice	Buckwheat flour, rice flour	Amylase	dov ^6^	[30]
GF bread	Rice	Rice flour	Cyclodextrinase	dov	[12,30]
GF bread	Rice, sorghum, maize	Rice, sorghum, maize flours	GO ^7^	dov	[12,30]
GF bread	Rice	Jasmine rice flour, pregelatinized tapioca starch	TGase	dov, TGase increased loaf volume and softened bread crumb.	[30,46,48]
GF bread	Oat	Oat flour	Tyrosinase, laccase, xylanase	dov, tyrosinase increased firmness of the dough, laccase and xylanase improved specific volume	
GF cake and muffin products	Rice	Rice flour, legume flour, chickpea flour, pea flour, lentil flour, bean flour	α-amylase, amyloglucosidase, trypsin, GO	dov	[32]
GF bread	Rice	Rice flour	0.01% GO 2% HPMC ^8^	↑ Final volume, smoother crumb	[12]
GF bread	Rice	Rice flour	1 U TGase/g	Improved crumb texture	[15]
GF bread	Rice	Rice flour	1.35 U of TGase/g rice flour protein 0.67% albumin 0.67% casein	↑ Final volume, less compact crumb	[12]
GF dough and bread	Rice	Rice flour	*Aspergillus oryzae* protease	↑ Viscosity, improved gas-holding capacity, volume improvements	
GF bread	Rice	Rice flour	Glutathione oxidase	↑ Elasticity and volume improved gas-holding capacity	[15]
GF bread	Rice	Rice flour	Microbial TGase HPMC	dov	[12]
GF bread	Rice	Rice flour	Proteases	Depending on protease amount added	

^1^ GF: gluten-free; ^2^ U: units; ^3^ TGase: transglutaminase; ^4^ ↑: results in an increase of the mentioned feature; ^5^ ↓: results in a decrease of the mentioned feature; ^6^ dov: dependent on variables; ^7^ GO: glucose oxidase; ^8^ HPMC: hydroxypropyl methylcellulose.

**Table 5:** Emulsifiers used in gluten-free baked goods.

**Food Product**	**Cereal(s) or Pseudo-Cereal(s) Used in the Product**	**Main Flour(s)**	**Emulsifiers**	**Technological Outcome**	**Reference**
GF ^1^ dough	Buckwheat	Buckwheat flour	DATEM^®^	dov ^2^	[12]
GF cheese bread	-	Cassava starch	DATEM^®^	dov	[50]
GF bread formulas	Rice	Rice flour	0.5% DATEM^®^ 0.5% (xanthan gum/guar)	Improved final product (with highest scores for texture acceptability)	[12]
GF bread	Rice	Rice flour, tigernut flour	DATEM^®^, xanthan gum, guar gum	dov	[32]
GF cake and muffin products	Rice, maize	Rice flour, maize flour	Lecithin	dov	

^1^ GF: gluten-free; ^2^ dov: dependent on variables.

## Data Availability

No new data were created or analyzed in this study. Data sharing is not applicable to this article.
